# Inflammatory biomarkers in sera of patients with intervertebral disc degeneration

**DOI:** 10.31744/einstein_journal/2019AO4637

**Published:** 2019-08-27

**Authors:** Luciano Miller Reis Rodrigues, Lilian Zerbinatti de Oliveira, Mariane de Barros Ribeiro da Silva, Camila de Melo Accardo, Adriana Braz Del Giglio, Maria Aparecida da Silva Pinhal

**Affiliations:** 1 Faculdade de Medicina do ABC, Santo André, SP, Brazil.; 2 Universidade Federal de São Paulo, São Paulo, SP, Brazil.; 3 Faculdade das Américas, São Paulo, SP, Brazil.

**Keywords:** Biomarkers, Proteoglycans, Glycosaminoglycans, Intervertebral disc degeneration

## Abstract

**Objective:**

To evaluate intervertebral disc levels of inflammatory factor (interleukin 6) and proteinase activity (cathepsin B) in patients with a degenerative disease and serum levels of interleukin 6, serum cathepsin B activity and hyaluronic acid biomarkers.

**Methods:**

We conducted immunohistochemistry studies of intervertebral discs to analyze interleukin 6 and cathepsin B levels of patients with degenerative disease and spine fracture (Control Group) and to measure hyaluronic acid, interleukin 6 and cathepsin B activity from sera of intervertebral disc degeneration patients, fracture patients, and healthy individuals.

**Results:**

Interleukin 6 and cathepsin B seem to be related with physiopathology of intervertebral disc degeneration, since the levels of both were higher in discs of patients with intervertebral disc degeneration. Interleukin 6 and cathepsin B do not represent good biomarkers of degenerative intervertebral disc disease, since the level of such compounds is increased in the plasma of patients with fractures.

**Conclusion:**

Hyaluronic acid can be a biomarker for intervertebral disc degeneration, because hyaluronic acid levels were higher only in sera of patients with intervertebral disc degeneration.

## INTRODUCTION

Intervertebral discs are composed of abundant cell matrix and low cell density. Two distinct regions in intervertebral discs can be characterize, *i.e* ., annulus fibrosus (AF) with high amounts of collagen and nucleus pulposus rich in proteoglycans.^(^^1^^)^ The principal proteoglycans found in intervertebral discs are aggrecan noncovalently attached to hyaluronic acid (HA). These proteoglycans' function allows compressive loads to intervertebral discs.^(^^2^^)^ Intervertebral disc degeneration (IVD) is associated with the loss of extracellular matrix (ECM) molecules, leading to alterations in the biochemical and biomechanical properties of the tissue.^(^^3^^-^^5^^)^

Enzymatic activity is believed to contribute to the degenerative process of IVD with increased collagen, proteoglycans and fibronectin fragmentation.^(^^6^^)^ Previous biochemical studies have shown the catabolism of these ECM molecules stimulated by several proteinases, such as metalloproteinases and collagenases.^(^^7^^,^^8^^)^

Cathepsins are cysteine proteases, a family of matrix degrading enzymes. Although published literature on cathepsins associated with IVD is scarce, these proteases seem to play an important role in the catabolic process of disc degeneration. Studies have demonstrated that cathepsin B (CatB) concentration in the cartilage of osteoarthritis patients is significantly higher than the levels found in normal tissues.^(^^9^^,^^10^^)^ Chu et al., suggested that CatB is released by synovial and inflammatory cells, and this releasing contributes to inflammation progression and cartilage destruction.^(^^11^^)^

Inflammatory cytokines are key players in the pathogenesis of IVD because they promote ECM disruption. Interleukin 6 (IL-6) can up-regulate matrix metalloproteinases and disintegrin and metalloproteinase with thrombospondin motifs (ADAMT) expression.^(^^12^^)^ Increased circulating levels of IL-6 have been reported in rheumatoid arthritis and osteoarthritis patients, which turn this cytokine a possible biomarker for disc degeneration.^(^^13^^-^^15^^)^

## OBJECTIVE

To evaluate intervertebral disc levels of possible inflammatory factors (interleukin 6 and cathepsin B) in patients with a degenerative disease, and compared them with healthy subjects (control). In addition, this study aim to investigate whether serum levels of interleukin 6, serum cathepsin B activity, or hyaluronic acid biomarkers reflect on intervertebral disc degeneration tissue status among patients with the intervertebral degenerative disease, control patients and patients with fractures.

## METHODS

### Study population

This study was approved by the Ethics Committee on Research involving Human of the *Faculdade de Medicina do ABC* (approval number 262/2008). Patients who signed the Informed Consent statement were included. All spinal cord injuries were in the lumbar region. Disc degeneration were found in L4/L5 and L5/S1, and fractures were observed in L1/L2, L2/L3 and L3/L4, all in the lumbar region. We obtained serum samples and intervertebral disc specimens from 83 patients who underwent primary lumbar discectomy with acute low back pain associated with radicular pain for less than 2 weeks. Patients' blood samples were collected during the follow-up of those who underwent surgery. We also obtained serum samples from 33 healthy subjects, without any spinal injury or inflammatory conditions, and who were used as controls. In addition, we also obtained intervertebral disc specimens from six patients who underwent surgery because of accidental fracture of the spine, and required disc removal. These patients had no spinal injury or previous inflammatory conditions and serum samples were taken from them. This group of patients was required to provide non-degenerated disc tissue for the immunohistochemistry analysis. The individuals enrolled in the study did not present any co-morbidity (hypertension, diabetes mellitus, chronic kidney disease or cancer), since such co-morbidities might increase the incidence of disc degeneration.

### Study design

It was a prospective study, and the subjects were selected from January 2015 to December 2017 at *Hospital Mário Covas* , in Santo André (SP), Brazil, and from the Orthopedic Surgery Department of the *Faculdade de Medicina do ABC* .

### Study limitations

The increase of the number of samples may enhance the statistical differences. The controlling of immunohistochemical reactions could not be performed with intervertebral disc tissues of healthy individuals because this study is not recommended from the ethical point of view. Therefore, as control tissue sample, we used tissues from patients who were affected by spinal fracture. However, a limitation is that these patients can present an acute inflammation process, and their chronic inflammation process does not present the common characteristics of those patients who suffer from IVD disease.

### Radiographic and clinical evaluation

All individuals were classified according to Pfirrmann's grading system for disc degeneration. A magnetic resonance imaging analysis was performed in all patients with a degenerative disc as well all spinal fracture patients (control). Only individuals classified as Pfirrmann grade III or IV, with one or two levels of degeneration, and nerve root compression at least in one level were included in the disc degeneration group, while Control Group was composed by individuals classified as Pfirrmann's grade I. Radiographies of the knee, shoulder, and hip of all individuals were also performed to assess the presence of degenerative changes in these joints. Exclusion criteria were defined as the presence of one or more of the following characteristics, systemic or inflammatory diseases, previous orthopedic surgery, ligament or muscular lesion, hypertension, hypercholesterolemia, diabetes, obesity (body mass index − BMI >30) or use of analgesic drugs during the preceding week.

### Serum hyaluronic acid levels

Sera from patients and controls were assayed for HA by a non-competitive and non-isotopic fluoroassay. This method is based on the affinity of specific proteins extracted from bovine cartilage (globular HA-binding region of the aggrecan and link protein) for HA, that detects HA between 0.2 and 500 *µ* g/L. This method has highly specificity and sensitivity (<0.2 *µ* g/L), and gives intra- and interassay coefficients of variation of 2 and 5%, and 3 and 14% in sera from normal subjects.^(^^16^^)^

### Serum interleukin 6 concentration

Serum levels of IL-6 were determined by the ELISA test using the kit Human IL-6 ELISA Ready-Set-Go!^®^ (eBioscience™ Inc, CA, USA) following the manufacturer's instructions.

### Cathepsin B activity

CatB activity was measured spectrofluorimetrically using the fluorogenic substrate Z-FR-MCA (Sigma, MO, USA) at 37°C. The fluorescence intensity was monitored in a microplate reader, with excitation and emission wavelengths set at 365 and 420nm. The assay was performed by preincubating the serum samples with the enzyme activator dithiothreitol (DTT) 2mM for 20 minutes and, then, by adding the irreversible inhibitor E-64, azocasein (Sigma, MO, USA), as previously described in study conducted by Almeida et al.^(^^9^^)^

### Immunohistochemical staining

Representative lumbar intervertebral degenerative disc regions were chosen based on the results of a hematoxylineosin staining study of the corresponding tissue sections. Three-micrometer-thick sections of formalin-fixed paraffin-embedded tissues were deparaffinized and rehydrated. The primary antibodies for IL-6 (Santa Cruz Biotechnology, CA, USA) and CatB (Calbiochem, USA) were diluted 1:350 and incubated overnight. A secondary biotinylated antibody (LSAB^®^, DakoCytomation^®^, Glostrup, Denmark) was applied for 30 minutes, and the slides were subsequently incubated with a peroxidase-labeled streptavidin complex (LSAB^®^, DakoCytomation^®^, Glostrup, Denmark) for additional 30 minutes. Sections were developed using 3,3'-diaminobenzidine (DAB) as the chromogen for 1 minute and, subsequently, counterstained with hematoxylin.

### Digital quantification

The slides were analyzed using a TS100 Nikon Eclipse^®^ light microscope to identify areas that best represented the immunostaining of IL-6 and CatB (hot spots). In each case, quantification of the degree of immunostaining was performed using a digital computer-assisted image analysis method. Photomicrographs (640×480 pixels) were obtained from non-coincident consecutive fields for each case at a magnification of 400x using a 4300 Nikon Coolpix^®^ digital camera adjusted to the same parameters. The obtained images were analyzed using the image processing and analysis system ImageLab^®^ (Softium Informática^®^, São Paulo, Brazil) adjusted to the micrometer scale. The images were performed at the same magnification fold (400x). The technique described by Matos et al.,^(^^17^^)^ was used to obtain the index of positive percentage of labeled cells (IP), the index of immunostaining intensity expression (ItE) and the index of expression (IE).

### Statistical analysis

The measures of the central tendency and value dispersion of the samples and the statistical tests for comparison between them were based on the data distribution type. The distributions were determined to be parametric by Kolmogorov-Smirnov testing. Each continuous variable value of the study was expressed by means and standard deviations. Absolute and relative frequencies were used for categorization. The analisys of variance (ANOVA) and Student's *t* test were used to compare the averages of the sample populations. The χ^2^ test was conducted to compared percentages. The significance level adopted was based on the chance of the occurrence of a type I error lower than 5% (p<0.05). For all analyses we used the Statistical Package for Social Science (SPSS), version 19.0 (SPSS Inc.; Illinois, USA).

## RESULTS

Table 1 shows the comparison of general characteristics (age, gender, smoking habits or manual labor) of patients with IVD degeneration, spine fracture and non-affected individuals (Control Group). No significant difference was seen among studied groups.

**Table 1:** General characteristics of the study population

	Controls	Disc degeneration	Fracture	p value
Number of patients	33	83	6	
Age	36.48±7.83	36.41±9.80	33.83±5.12	0.794
Gender (female/male)	18/15	32/51	2/4	0.260
Smokers	5 (15.2)	20 (24.1)	1 (16.7)	0.511
Manual laborers	6 (18.2)	31 (37.3)	2 (33.3)	0.136

Results expressed as n, mean±standard deviation or n (%).

### Immunohistochemistry analysis of intervertebral disc tissue

**Figure 1:**
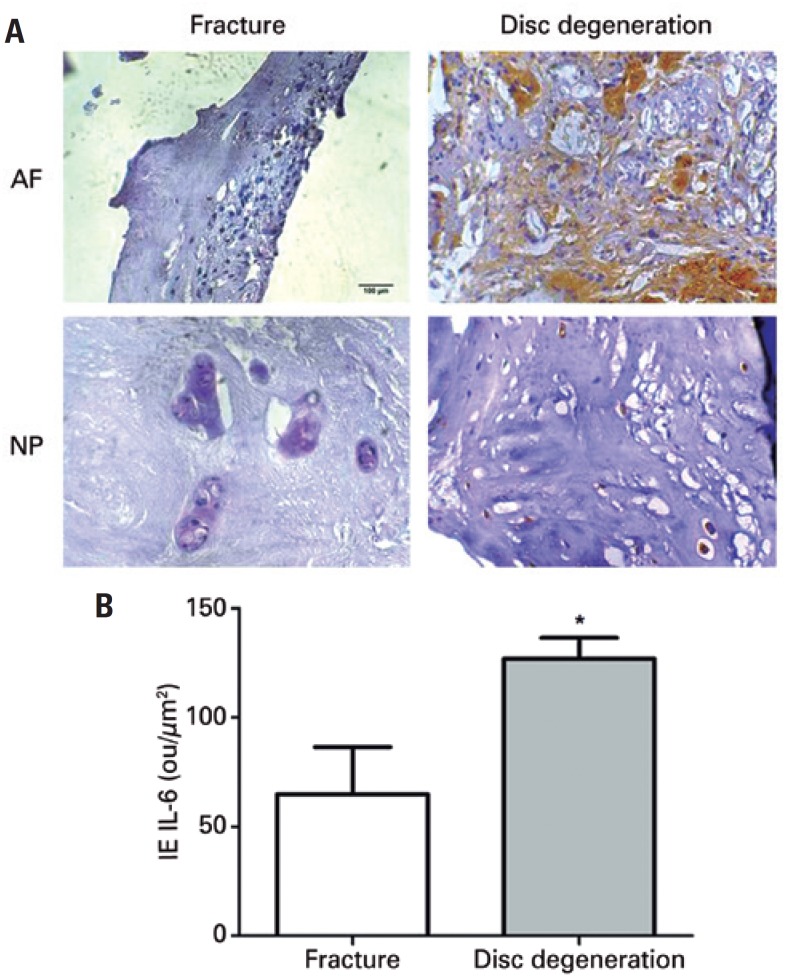
Immunohistochemistry of Interleukin 6 (IL-6). (A) Intervertebral disc labeled with anti-IL-6 antibody. Disc degeneration refers to patient with intervertebral disc degeneration. Fracture refers to patient with spine fracture (Control Group). (B) Quantification of immunohistochemistry. The average and standard deviation were represented as bars and lines * p<0.05. AF: annulus fibrosus; NP: nucleus pulposus; IE: index expression.

**Figure 2:**
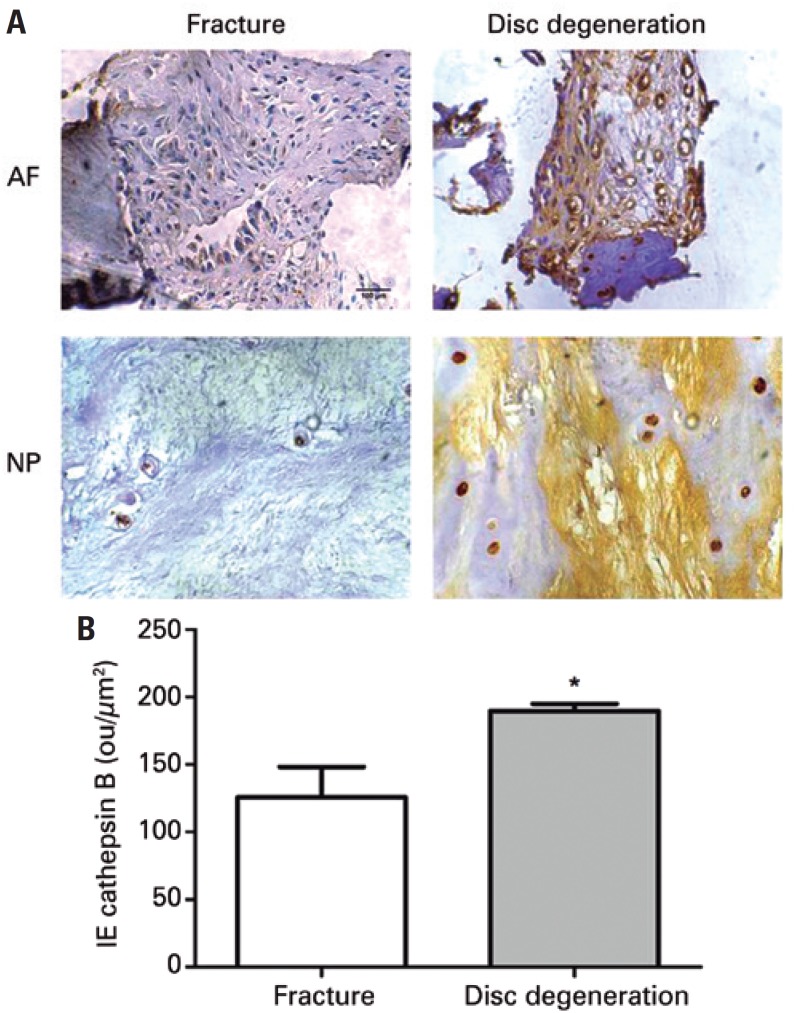
Immunohistochemistry of cathepsin B. (A) Intervertebral disc labeled with anti-cathepsin B antibody. Disc degeneration refers to patient with intervertebral disc degeneration. Fracture refers to patient with spine fracture (Control Group). (B) Quantification of immunohistochemistry. The average and standard deviation were represented as bars and lines * p<0.05. AF: annulus fibrosus; NP: nucleus pulposus; IE: index expression.

The protein expression of IL-6 in degenerative disc tissues and in discs of patients who had suffered accidental spine fracture was obtained by digital quantification of immunohistochemistry reactions. Immunoreactivity for IL-6 was more intense in the AF and nucleus pulposus (NP) of degenerative discs than in the spine fracture ( Figure 1A ). Annulus fibrosus from degenerative discs presented more intense immunoreactivity for IL-6 in the ECM compared with the labeling in fibroblasts. Strong immunoreactivity for IL-6 was also present in the chondrocytes of NP from degenerative discs, whilst no labeling can be observed in the ECM ( Figure 1A ). The IE of IL-6 was significantly higher in the degenerative discs compared with the fracture IVD ( Figure 1B ). The expression of CatB was also investigated in the degenerative intervertebral discs and spine fracture using immunohistochemistry. The test demonstrated a low-intensity CatB labeling in the AF and NP of fracture discs ( Figure 2A ), whereas IVD with degeneration presented intense immunoreactivity ( Figure 2A ). Intense CatB labeling both in chondrocytes and ECM was observed in the NP from degenerative discs ( Figure 2A ). Therefore, protein intensity of CatB was significantly higher in the degenerative IVD compared to the fracture discs ( Figure 2B ).

### Circulating levels of interleukin-6, cathepsin B and hyaluronic acid

**Figure 3:**
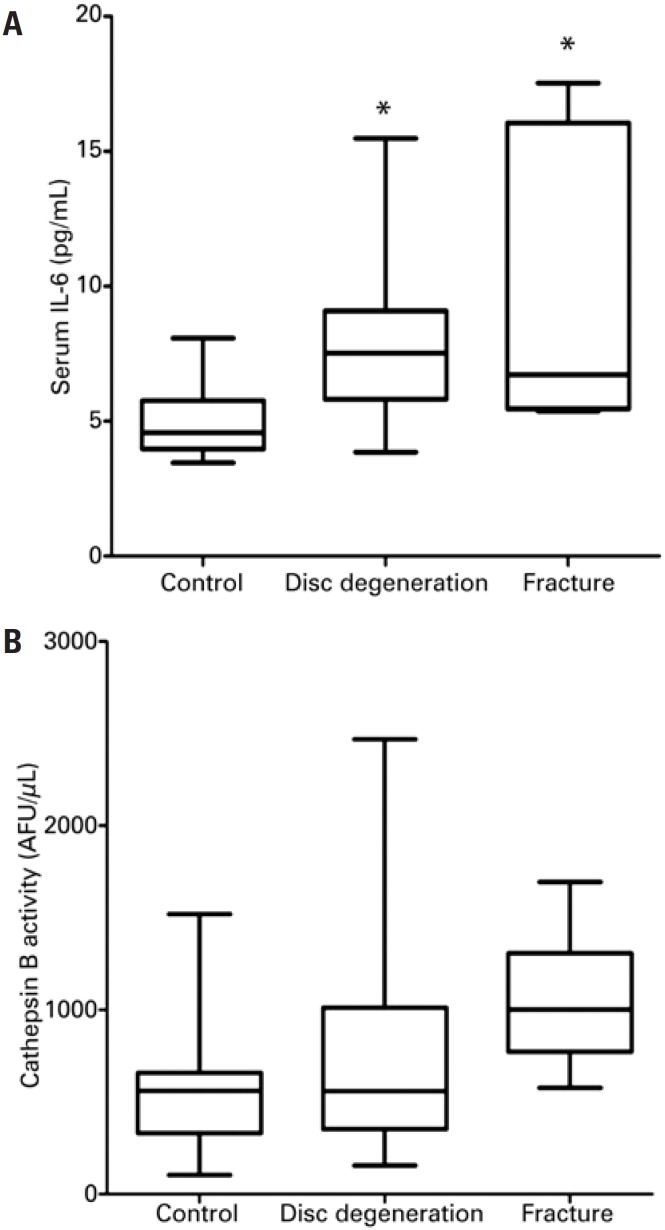
Serum levels of interleukin-6 (IL-6) and cathepsin B activity. Control refers to healthy patient. Disc degeneration refers to patient with intervertebral disc degeneration. Fracture refers to patient with spine fracture * p<0.05. AF: annulus fibrosus.

**Figure 4:**
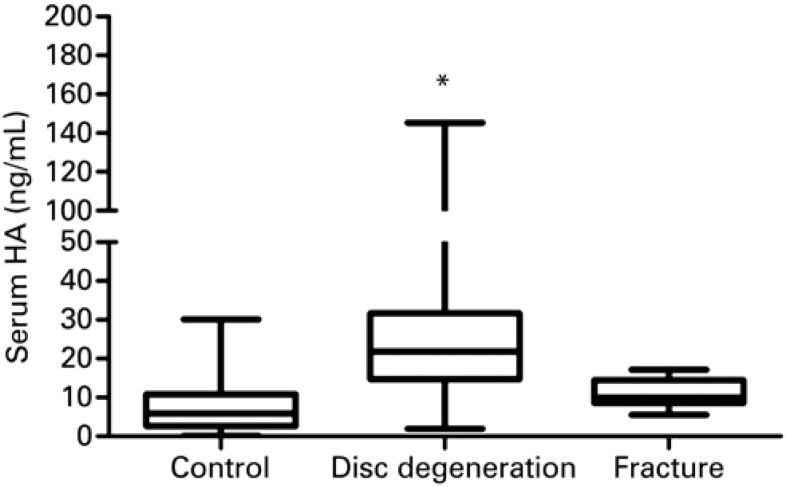
Serum levels of hyaluronic acid. Control refers to healthy patient. Disc degeneration refers to patient with intervertebral disc degeneration. Fracture refers to patient with spine fracture * p<0.05. HA: hyaluronic acid.

Circulating levels of IL-6 in disc degeneration and fracture patients were statistically higher compared to the Control Group (p<0.001), as demonstrated in figure 3A . There was no significant difference comparing IL-6 levels between IVD and fracture patients ( Figure 3A ). Measurement of CatB activity in sera of patients with IVD, intervertebral disc fracture and non-affected individuals (control), demonstrated that there was no significant difference among groups ( Figure 3B ). However, serum levels of HA were statistically higher in patients with IVD (p<0.001) compared with Control Group ( Figure 4 ), whilst, no significant difference was observed in the circulating HA levels between fracture patients and Control Group ( Figure 4 ).

## DISCUSSION

CatB is a protease which is believed to have an important role in the degradation of cartilage ECM proteins, such as aggrecan and collagen.^(^^10^^,^^12^^,^^18^^)^ Enhanced expression of CatB has been reported in chondrocytes of osteoarthritis patients.^(^^19^^)^ In addition, intervertebral degenerated cells has been believed to be responsible of secretion pro-inflammatory cytokines, including IL-6.^(^^20^^)^ The persistent proinflammatory activity of IL-6 favors mononuclear cell accumulation at the site of injury, angio-proliferation and antiapoptotic functions of T cells.^(^^21^^-^^23^^)^

Data suggest that both IL-6 and CatB are close related to physiopathology of IVD. In our study, CatB protein expression was significantly increased in degenerative disc tissues compared with fracture discs. Intense immunoreactivity for CatB was found in the ECM of degenerative disc specimens, suggesting that the acidic microenvironment is likely to cause inflammatory process favored by CatB secretion. Also, IL-6 protein levels were higher compared with control intervertebral discs.

No significant difference was found concerning CatB activity and IL-6 among groups when sera were analyzed. Circulating IL-6 and CatB concentrations in patients with spine fracture were also significantly higher compared with the Control Group, showing that increase of IL-6 and CatB in serum is not specific to IVD. The increased serum concentration of IL-6 and CatB in fracture patients may be due to the acute inflammation caused by the fracture, especially if we consider that peripheral blood was collected just after trauma.

Quantitative analyses of serum HA are useful in the diagnosis of several inflammatory diseases.^(^^16^^,^^24^^,^^25^^)^ Extracellular matrix degradation and turnover result in the release of HA and HA fragments into the systemic circulation.^(^^26^^)^ Different from patients who underwent anti-inflammatory treatment, low levels of HA were found in the circulation.^(^^23^^,^^26^^)^

We may suggest that increased levels of HA might indicate a degeneration process. However, other studies are necessary to confirm this conclusion, considering that our study represent preliminary results.

Degradation of ECM components on the IVD process is modulated by several proteolytic enzymes of which matrix metalloproteinases (MMPs) and aggrecanases play a critical role in the activation of IL-6, IL-8, cyclo-oxygenase-2 (COX2), MMP1/13 and Toll Like Receptors (TLR2).^(^^27^^,^^28^^)^

Several strategies aiming the regeneration of the intervertebral disc have been used lately. These strategies, with promising results, involve cellular therapies, as well as biomaterials such as the HA scaffold.^(^^28^^,^^29^^)^

Our results may contribute to further studies and may promote the use of these target molecules for the IVD process.

## CONCLUSION

Serum hyaluronic acid levels are significantly higher in patients with intervertebral disc degeneration than in non-affected patients, and those who had spine fracture. Data confirm that interleukin 6 and cathepsin B are possible related to the physiopathology of intervertebral disc degeneration, however they do not represent a biomarker in serum.

## References

[1] 1. Martins DE, Medeiros VP, Wajchenberg M, Paredes-Gamero EJ, Lima M, Reginato RD, et al. Changes in human intervertebral disc biochemical composition and bony end plates between middle and old age. PLoS One. 2018;13(9):e0203932. 10.1371/journal.pone.0203932 PMC6144914 30226874

[2] 2. Sivan SS, Wachtel E, Roughley P. Structure, function, aging and turnover of aggrecan in the intervertebral disc. Biochim Biophys Acta. 2014; 1840(10):3181-9. Review. 10.1016/j.bbagen.2014.07.013 25065289

[3] 3. Ji ML, Lu J, Shi PL, Zhang XJ, Wang SZ, Chang Q, et al. Dysregulated miR-98 contributes to extracellular matrix degradation by targeting IL-6/STAT3 signaling pathway in human intervertebral disc degeneration. J Bone Miner Res. 2016;31(4):900-9. 10.1002/jbmr.2753 26587789

[4] 4. Wuertz K, Vo N, Kletsas D, Boos N. Inflammatory and catabolic signalling in intervertebral discs: the roles of NF-kappaB and MAP kinases. Eur Cell Mater. 2012;23:103-19; discussion 19-20. 10.22203/ecm.v023a08 22354461

[5] 5. Gruber HE, Hanley Jr. EN. Do We Need Biomarkers for Disc Degeneration? Biomark Insights. 2006;1:131:3. PMC2716792 19690643

[6] 6. Urban JP, Roberts S. Degeneration of the intervertebral disc. Arthritis Res Ther. 2003;5(3):120-30. Review. 10.1186/ar629 PMC165040 12723977

[7] 7. Wang WJ, Yu XH, Wang C, Yang W, He WS, Zhang SJ, et al. MMPs and ADAMTSs in intervertebral disc degeneration. Clin Chim Acta. 2015;448:238-46. Review. 10.1016/j.cca.2015.06.023 26162271

[8] 8. Gruber HE, Ingram JA, Hoelscher GL, Zinchenko N, Norton HJ, Hanley EN Jr. Constitutive expression of cathepsin K in the human intervertebral disc: new insight into disc extracellular matrix remodeling via cathepsin K and receptor activator of nuclear factor-kappaB ligand. Arthritis Res Ther. 2011;13(4):R140. 10.1186/ar3454 PMC3239383 21880134

[9] 9. Almeida PC, Nantes IL, Chagas JR, Rizzi CC, Faljoni-Alario A, Carmona E, et al. Cathepsin B activity regulation. Heparin-like glycosaminogylcans protect human cathepsin B from alkaline pH-induced inactivation. J Biol Chem. 2001;276(2):944-51. 10.1074/jbc.M003820200 11016923

[10] 10. Ha SD, Martins A, Khazaie K, Han J, Chan BM, Kim SO. Cathepsin B is involved in the trafficking of TNF-alpha-containing vesicles to the plasma membrane in macrophages. J Immunol. 2008;181(1):690-7. 10.4049/jimmunol.181.1.690 18566436

[11] 11. Chu SC, Yang SF, Tzang BS, Hsieh YS, Lue KH, Lu KH. Cathepsin B and cystatin C play an inflammatory role in gouty arthritis of the knee. Clin Chim Acta. 2010;411(21-22):1788-92. 10.1016/j.cca.2010.07.037 20699092

[12] 12. Dudek M, Yang N, Ruckshanthi JP, Williams J, Borysiewicz E, Wang P, et al. The intervertebral disc contains intrinsic circadian clocks that are regulated by age and cytokinesand linked to degeneration. Ann Rheum Dis. 2017;76(3):576-84. 10.1136/annrheumdis-2016-209428 PMC5446006 27489225

[13] 13. Gabay C. Interleukin-6 and chronic inflammation. Arthritis Res Ther. 2006;8 Suppl 2:S3. Review. 10.1186/ar1917 PMC3226076 16899107

[14] 14. Weber KT, Alipui DO, Sison CP, Bloom O, Quraishi S, Overby MC, et al. Serum levels of the proinflammatory cytokine interleukin-6 vary based on diagnoses in individuals with lumbar intervertebral disc diseases. Arthritis Res Ther. 2016;18:3. 10.1186/s13075-015-0887-8 PMC4718017 26743937

[15] 15. Suzuki S, Fujita N, Fujii T, Watanabe K, Yagi M, Tsuji T, et al. Potential involvement of the IL-6/JAK/STAT3 pathway in the pathogenesis of intervertebral disc degeneration. Spine (Phila Pa 1976). 2017;42(14):E817-24. 10.1097/BRS.0000000000001982 27879577

[16] 16. Martins JR, Passerotti CC, Maciel RM, Sampaio LO, Dietrich CP, Nader HB. Practical determination of hyaluronan by a new noncompetitive fluorescence-based assay on serum of normal and cirrhotic patients. Anal Biochem. 2003;319(1):65-72. 10.1016/s0003-2697(03)00251-3 12842108

[17] 17. Matos LL, Stabenow E, Tavares MR, Ferraz AR, Capelozzi VL, Pinhal MA. Immunohistochemistry quantification by a digital computer-assisted method compared to semiquantitative analysis. Clinics (Sao Paulo). 2006;61(5):417-24. 10.1590/s1807-59322006000500008 17072439

[18] 18. Ruettger A, Schueler S, Mollenhauer JA, Wiederanders B. Cathepsins B, K, and L are regulated by a defined collagen type II peptide via activation of classical protein kinase C and p38 MAP kinase in articular chondrocytes. J Biol Chem. 2008;283(2):1043-51. 10.1074/jbc.M704915200 17991740

[19] 19. Li Y, Li K, Han X, Mao C, Zhang K, Zhao T, et al. The imbalance between TIMP3 and matrix-degrading enzymes plays an important role in intervertebral disc degeneration. Biochem Biophys Res Commun. 2016;469(3):507-14. 10.1016/j.bbrc.2015.12.020 26686417

[20] 20. Kang JD, Georgescu HI, McIntyre-Larkin L, Stefanovic-Racic M, Donaldson WF 3rd, Evans CH. Herniated lumbar intervertebral discs spontaneously produce matrix metalloproteinases, nitric oxide, interleukin-6, and prostaglandin E2. Spine (Phila Pa 1976). 1996;21(3):271-7. 10.1097/00007632-199602010-00003 8742201

[21] 21. Niki Y, Takeuchi T, Nakayama M, Nagasawa H, Kurasawa T, Yamada H, et al. Clinical significance of cartilage biomarkers for monitoring structural joint damage in rheumatoid arthritis patients treated with anti-TNF therapy. PLoS One. 2012;7(5):e37447. 10.1371/journal.pone.0037447 PMC3357428 22629396

[22] 22. Igarashi A, Kikuchi S, Konno S. Correlation between inflammatory cytokines released from the lumbar facet joint tissue and symptoms in degenerative lumbar spinal disorders. J Orthop Sci. 2007;12(2):154-60. 10.1007/s00776-006-1105-y 17393271

[23] 23. Podichetty VK. The aging spine: the role of inflammatory mediators in intervertebral disc degeneration. Cell Mol Biol (Noisy-le-grand). 2007;53(5):4-18. Review. 17543240

[24] 24. Jiang D, Liang J, Noble PW. Hyaluronan as an immune regulator in human diseases. Physiol Rev. 2011;91(1):221-64. Review. 10.1152/physrev.00052.2009 PMC3051404 21248167

[25] 25. Yagmur E, Koch A, Haumann M, Kramann R, Trautwein C, Tacke F. Hyaluronan serum concentrations are elevated in critically ill patients and associated with disease severity. Clin Biochem. 2012;45(1-2):82-7. 10.1016/j.clinbiochem.2011.10.016 22085533

[26] 26. Pitsillides AA, Will RK, Bayliss MT, Edwards JC. Circulating and synovial fluid hyaluronan levels. Effects of intraarticular corticosteroid on the concentration and the rate of turnover. Arthritis Rheum. 1994;37(7):1030-8. 10.1002/art.1780370708 8024612

[27] 27. Bachmeier BE, Nerlich A, Mittermaier N, Weiler C, Lumenta C, Wuertz K, et al. Matrix metalloproteinase expression levels suggest distinct enzyme roles during lumbar discherniation and degeneration. Eur Spine J. 2009; 18(11):1573-86. 10.1007/s00586-009-1031-8 PMC2899407 19466462

[28] 28. van Uden S, Silva-Correia J, Oliveira JM, Reis RL. Current strategies for treatment of intervertebral disc degeneration: substitution and regeneration possibilities. Biomater Res. 2017;21:22. Review. 10.1186/s40824-017-0106-6 PMC5651638 29085662

[29] 29. Fernandez-Moure J, Moore CA, Kim K, Karim A, Smith K, Barbosa Z, et al. Novel Therapeutic strategies for degenerative disc disease: review of cell biology and intervertebral disc cell therapy. SAGE Open Med. 2018; 6:2050312118761674. Review. 10.1177/2050312118761674 PMC5858682 29568524

