# Supplementary material for: Inflammatory biomarkers in sera of patients with intervertebral disc degeneration
Source: Einstein (Sao Paulo). 2019 Aug 27;17(4):eAO4637. doi: 10.31744/einstein_journal/2019AO4637 (PMC6711751; doi:10.31744/einstein_journal/2019AO4637)
Supplement: Translation

# Translation: Biomarcadores inflamatórios no plasma de pacientes com degeneração do disco intervertebral

**Authors:** Luciano Miller Reis Rodrigues, Lilian Zerbinatti de Oliveira, Mariane de Barros Ribeiro da Silva, Camila de Melo Accardo, Adriana Braz Del Giglio, Maria Aparecida da Silva Pinhal

**Affiliations:** 1 Faculdade de Medicina do ABC, Santo André, SP, Brasil.; 2 Universidade Federal de São Paulo, São Paulo, SP, Brasil.; 3 Faculdade das Américas, São Paulo, SP, Brasil.

**Keywords:** Biomarcadores, Proteoglicanas, Glicosaminoglicanos, Degeneração do disco intervertebral

## Abstract

**Objetivo:**

Avaliar os níveis de fatores inflamatórios nos discos intervertebrais (interleucina 6) e proteinase (catepsina B) em pacientes com doença degenerativa de disco intervertebral, além de verificar os níveis séricos de interleucina 6, ácido hialurônico e atividade sérica da catepsina B.

**Métodos:**

Foi realizado exame imuno-histoquímica dos discos intervertebrais de pacientes com doença degenerativa e fratura da coluna (Grupo Controle) e análise do plasma de pacientes com doença degenerativa de disco intervertebral. Como controle, foram utilizados plasma de pacientes com fraturas, além de indivíduos saudáveis.

**Resultados:**

Interleucina 6 e catepsina B sugerem relação com a fisiopatologia da doença degenerativa de disco intervertebral, uma vez que os níveis de ambos foram maiores nos discos de pacientes com doença degenerativa de disco intervertebral. Interleucina 6 e catepsina B não representam bons biomarcadores da doença degenerativa do disco intervertebral, já que também encontram níveis aumentados em plasma de pacientes com fratura.

**Conclusão:**

O ácido hialurônico é um possível biomarcador de doença degenerativa de disco intervertebral, porque os níveis de ácido hialurônico foram maiores apenas em plasma de pacientes com doença degenerativa de disco intervertebral.

## INTRODUÇÃO

Os discos intervertebrais são compostos por matriz celular abundante e baixa densidade celular. Há possibilidade de caracterização de duas regiões distintas nos discos intervertebrais: anel fibroso, com altas quantidades de colágeno, e núcleo pulposo (NP), rico em proteoglicanos.^(^^1^^)^Os principais proteoglicanos encontrados nos discos intervertebrais são agrecan aderidos de forma não covalente ao ácido hialurônico (AH). Sua função é permitir cargas compressivas nos discos intervertebrais.^(^^2^^)^

A degeneração do disco intervertebral (DDI) é associada à perda de moléculas da matriz extracelular (MEC), e a alterações nas propriedades bioquímicas e biomecânicas do tecido.^(^^3^^-^^5^^)^

É aceito que a atividade enzimática contribui para o processo degenerativo da DIV com aumento do colágeno, proteoglicanos e fragmentação da fibronectina.^(^^6^^)^Estudos bioquímicos prévios evidenciaram o catabolismo dessas moléculas de MEC por várias proteinases, como metaloproteinases e colagenases.^(^^7^^,^^8^^)^

As catepsinas são proteases de cisteína, uma família de enzimas que degradam a matriz e, embora a literatura sobre elas na DDI seja escassa, essas proteases possivelmente desempem papel importante no processo catabólico da degeneração discal. Estudos demonstraram que a concentração de catepsina B (CatB) na cartilagem de pacientes com osteoartrite é significativamente maior do que no tecido normal.^(^^9^^,^^10^^)^Chu et al., sugeriram que a CatB é liberada pelas células sinoviais e inflamatórias, contribuindo para a progressão da inflamação e a destruição da cartilagem.^(^^11^^)^

As citocinas inflamatórias são elementos-chave na patogênese da DDI, promovendo a ruptura do MEC. A interleucina 6 (IL-6) pode regular positivamente as metaloproteinases da matriz e a expressão de *A disintegrin and metalloproteinase with thrombospondin motifs* (ADAMT).^(^^12^^)^Os níveis circulantes aumentados de IL-6 foram encontrados em pacientes com artrite reumatoide e osteoartrite, tornando essa citocina um possível biomarcador para degeneração discal.^(^^13^^-^^15^^)^

## OBJETIVO

Avaliar os níveis de disco intervertebral de possíveis fatores inflamatórios (interleucina 6 e catepsina B) em pacientes com doença degenerativa comparados a indivíduos saudáveis (controle) e investigar se os níveis séricos de interleucina 6, a atividade sérica de catepsina B ou os biomarcadores de ácido hialurônico refletem no status do tecido com degeneração do disco intervertebral entre pacientes com degeneração do disco intervertebral, controles e com fratura.

## METÓDOS

### População do estudo

O presente estudo foi aprovado pelo Comitê de Ética em Pesquisa com seres humanos da Faculdade de Medicina do ABC (parecer 262/2008 do Comitê de Ética em Pesquisa). Os pacientes foram incluídos após assinatura do Termo de Consentimento. Todas as lesões na medula espinhal estavam localizadas na região lombar. O nível de degeneração discal foi em L4/L5 e L5/S1, e o nível de fratura foi L1/L2, L2/L3 e L3/L4, sendo todos na coluna lombar. Foram obtidas amostras de plasma e dos discos intervertebrais de 83 pacientes submetidos à discectomia lombar primária, com dor lombar aguda associada à dor radicular por menos de 2 semanas. Amostras de sangue dos pacientes foram coletadas durante o acompanhamento dos pacientes que necessitaram de cirurgia. Além disso, também obtivemos amostras de plasma de 33 indivíduos saudáveis, sem qualquer lesão medular ou condição inflamatória, que compuseram o Grupo Controle. Além disso, espécimes do disco intervertebral também foram obtidos de seis pacientes submetidos à cirurgia por fratura acidental da coluna vertebral, o que exigiu a remoção do disco. Tais pacientes não apresentavam lesão medular e nem condições inflamatórias prévias e forneceram amostras de plasma. Este grupo de pacientes foi obrigado a fornecer tecido discal não degenerado para a análise imuno-histoquímica. Os sujeitos inscritos no estudo não apresentaram qualquer comorbilidade (hipertensão, *diabetes mellitus* , doença renal crônica ou cancro), uma vez que é sabido que tais comorbidades poderiam aumentar a incidência de degeneração discal.

### Desenho do estudo

Estudo prospectivo em que os sujeitos foram selecionados no período de janeiro de 2015 a dezembro de 2017 no Hospital Mário Covas, em Santo André (SP), e no Departamento de Cirurgia Ortopédica da Faculdade de Medicina do ABC.

### Limitação do estudo

O aumento do número de amostras pode beneficiar as diferenças estatísticas. O controle das reações imuno-histoquímicas não pode ser realizado com os tecidos do disco intervertebral de indivíduos saudáveis, pois não é recomendado do ponto de vista ético. Assim, como amostra de tecido controle, foram utilizados pacientes afetados por fratura da coluna vertebral. No entanto, esses pacientes são conhecidos por apresentarem processo agudo de inflamação, mas não por terem um processo de inflamação crônica, que é uma característica comum em pacientes que sofrem de doença DDI.

### Avaliação radiográfica e clínica

Todos os indivíduos foram classificados de acordo com o sistema de classificação de Pfirrmann para degeneração discal. A análise por ressonância magnética foi realizada em todos os pacientes com um disco degenerativo, bem como com todos aqueles apresentando fratura da coluna vertebral (controle). Apenas indivíduos classificados como Pfirrmann grau III ou IV, com 1 ou 2 níveis de degeneração, e compressão de raiz nervosa em pelo menos um nível, foram incluídos no grupo de degeneração discal, enquanto o Grupo Controle foi composto por indivíduos classificados como Pfirrmann grau I. As radiografias de joelho, ombro e quadril de todos os indivíduos também foram conduzidas para avaliar a presença de alterações degenerativas nessas articulações. Os critérios de exclusão foram definidos também com presença de uma ou mais das seguintes características: doenças sistêmicas ou inflamatórias, cirurgia ortopédica prévia, lesão ligamentar ou muscular, hipertensão, hipercolesterolemia, diabetes, obesidade (índice de massa corporal − IMC >30) ou uso de analgésicos durante a semana anterior ao procedimento.

### Níveis da dosagem sérica do ácido hialurônico

O plasma dos pacientes e controles foram testados quanto ao AH por ensaio fluoro não competitivo e não isotópico. Este método é baseado na afinidade de ligação de uma proteína globular específica, proteína de ligação, responsável pela interação entre a molécula de AH com o esqueleto proteico do agrecam, que detecta quantidades de AH entre 0,2 a 500 *μ* g/L. Esse método é muito específico e sensível (<0,2 *µ* g/L), dando coeficientes de variação intra e interensaio de 2 a 5% e 3 a 14%, respectivamente, em plasma de indivíduos normais.^(^^16^^)^

### Concentração séricas de interleucina 6

Os níveis de IL-6 foram determinados por ELISA utilizando *kit* humano IL-6 ELISA Ready-Set-Go!^®^(eBioscience™ Inc, CA, EUA), conforme instruções do fabricante.

### Atividade catepsina B

A CatB foi quantificada por fluorescência utilizando o substrato Z-FR-MCA fluorogênico (Sigma, MO, EUA) em 37°C. A intensidade fluorescente foi monitorada por leitor ( *microplate reader* ) com excitação e emissão de ondas com frequência estabelecida entre 365 e 420nm, respectivamente. O ensaio foi pré-incubado e as amostras séricas com enzima ativadora de ditiotreitol (DTT) 2mM por 20 minutos. Após, foi adicionado inibidor irreversível E-64, azocaseína (Sigma, MO, EUA), como previamente descrito por Almeida et al.^(^^9^^)^

### Coloração imuno-histoquímica

Região lombar de discos intervertebrais degenerados foram escolhidas com base nos resultados da coloração por hematoxilina-eosina das secções dos tecidos. Secções de 3 *µ* m de espessura de tecidos embebidos em parafina fixados em formalina foram desparafinizadas e reidratadas. Os anticorpos primários para IL-6 (Santa Cruz Biotechnology, CA, EUA) e CatB (Calbiochem, EUA) foram diluídos a 1:350 e incubados *overnight* . Um anticorpo biotinilado secundário (LSAB^®^, DakoCytomation^®^, Glostrup, Dinamarca) foi aplicado durante 30 minutos, e as lâminas foram subsequentemente incubadas com complexo de estreptavidina marcado com peroxidase (LSAB^®^, DakoCytomation^®^, Glostrup, Dinamarca) por 30 minutos adicionais. As secções foram desenvolvidas usando 3,3'-diaminobenzidina (DAB) como o cromógeno durante 1 minuto e posteriormente contracoradas com hematoxilina.

### Quantificação digital

As placas foram analisadas utilizando um microscópio Nikon Eclipse^®^TS100 para identificar as áreas que melhor representassem coloração imuno de IL-6 e CatB (locais de calor). Em cada caso, a quantificação do grau de imunocoloração foi realizada usando método digital de análise de imagem assistida por computador. Fotomicrografias (640×480 *pixels* ) foram obtidas de campos consecutivos não coincidentes para cada caso, com ampliação de 400 vezes por câmera digital Nikon Coolpix^®^4300 ajustada aos mesmos parâmetros. As imagens resultantes foram analisadas no sistema de processamento e análise de imagens ImageLab^®^(Softium Informática^®^, São Paulo, Brasil) ajustado à escala do micrômetro. As imagens foram confeccionadas na mesma escala de ampliação (400 vezes). O índice de percentagem (IP) positiva de células marcadas, o índice de expressão da intensidade de imunocoloração (ItC) e o índice de expressão (IE) foram obtidos, conforme descrito por Matos et al.^(^^17^^)^

### Análise estatística

As medidas de tendência central e dispersão de valor das amostras e os testes estatísticos para comparação entre elas foram baseadas no tipo de distribuição de dados. As distribuições foram determinadas como paramétricas pelo teste de Kolmogorov-Smirnov. Cada valor de variável contínua obtido no estudo foi expresso como média e desvio padrão. Utilizaram-se frequências absolutas e relativas para categorização. Para comparar as médias das populações amostrais, utilizaram-se o teste de análise de variância ANOVA e o teste *t* de *Student* . As comparações entre percentagens foram conduzidas pelo teste do χ^2^. Adotou-se nível de significância baseado na chance de ocorrência de erro tipo I menor que 5% (p<0,05). Para todas as análises, adotou-se o *Statistical Package for Social Science* (SPSS), versão 19.0 (SPSS Inc.; Illinois, EUA).

## RESULTADOS

A tabela 1 mostra a comparação das características gerais dos pacientes com degeneração de DDI, fratura de coluna e indivíduos não afetados (Grupo Controle), demonstrando que não houve diferença significativa entre os grupos incluídos no estudo em relação a idade, gênero, tabagismo ou trabalho manual.

**Tabela 1:** Características gerais da população do estudo

	Controles	Degeneração do disco	Fratura	Valor de p
Pacientes	33	83	6	
Idade	36,48±7,83	36,41±9,80	33,83±5,12	0,794
Gênero (feminino/masculino)	18/15	32/51	2/4	0,260
Tabagistas	5 (15,2)	20 (24,1)	1 (16,7)	0,511
Trabalhadores que realizavam atividade manual	6 (18,2)	31 (37,3)	2 (33,3)	0,136

Resultados expressos por n, média±desvio padrão ou n (%).

### Análise inmuno-histoquímica de tecido do disco invertebral

A expressão proteica da IL-6 em tecidos discais degenerativos e em discos de pacientes com fratura acidental da coluna foi obtida por quantificação digital de reações imuno-histoquímicas. A imunorreatividade para a IL-6 foi mais intensa no ânulo fibroso (AF) e no NP dos discos degenerativos do que na fratura da coluna ( Figura 1A ). Pode-se demonstrar que o AF dos discos degenerativos apresentou imunorreatividade mais intensa para a IL-6 na MEC em comparação com a marcação nos fibroblastos. Imunorreatividade forte para IL-6 também estava presente nos condrócitos de NP de discos degenerativos, enquanto nenhuma marcação pôde ser observada nas MEC ( Figura 1A ). O IE da IL-6 foi significativamente maior nos discos degenerativos em comparação com a DDI da fratura ( Figura 1B ). A expressão de CatB também foi investigada nos discos intervertebrais degenerativos e na fratura da coluna usando imuno-histoquímica. A expressão demonstrou marcação CatB de baixa intensidade no AF e NP dos discos de fratura ( Figura 2A ), enquanto que discos intervertebrais com degeneração apresentaram imunoreatividade intensa ( Figura 2A ). A marcação intensa de CatB tanto nos condrócitos quanto na MEC foi observada na NP de discos degenerativos ( Figura 2A ). Assim, a intensidade proteica da CatB foi significativamente maior na DDI degenerativa em comparação com os discos de fratura ( Figura 2B ).

**Figura 1:** Imuno-histoquímica da interleucina 6 (IL-6). (A) Disco intervertebral marcado com anticorpo anti-IL-6. Degeneração do disco se refere a paciente com degeneração do disco intervertebral. Fratura abrange paciente com fratura de coluna vertebral (Grupo Controle). (B) Quantificação de imuno-histoquímica. A média e o desvio padrão foram representados como barras e linhas, respectivamente * p<0,05. AF: ânulo fibroso; NP: núcleo pulposo; IE: índice de expressão.

**Figura 2:** Imunohistoquímica da catepsina B. (A) Disco intervertebral marcado com anticorpo anti-catepsina B. Degeneração do disco se refere a paciente com degeneração do disco intervertebral. Fratura abrange paciente com fratura de coluna vertebral (Grupo Controle). (B) Quantificação de imuno-histoquímica. A média e o desvio padrão foram representados como barras e linhas, respectivamente * p<0,05. AF: ânulo fibroso; NP: núcleo pulposo; IE: índice de expressão.

### Níveis de circulação de interleucina-6, catepsina B e ácido hialurônico

Os níveis circulantes de IL-6 nos pacientes com degeneração discal e fratura foram estatisticamente maiores em comparação ao Grupo Controle (p<0,001), conforme demonstrado na figura 3A . Não houve diferença significativa comparando os níveis de IL-6 entre DDI e pacientes com fratura ( Figura 3A ). A mensuração da atividade de CatB em plasma de pacientes com DDI, fratura do disco intervertebral e indivíduos não afetados (Grupo Controle) demonstrou inexistência de diferença significativa entre os grupos ( Figura 3B ). No entanto, os níveis séricos de AH foram estatisticamente maiores nos pacientes com DDI (p<0,001) comparados ao controle ( Figura 4 ), e não houve diferença significativa nos níveis circulantes de AH entre os pacientes com fratura e Grupo Controle ( Figura 4 ).

**Figura 3:** Níveis séricos de interleucina 6 (IL-6) e atividade da catepsina B. Controle se refere a paciente saudável. Degeneração do disco se refere a paciente com degeneração do disco intervertebral. Fratura abrange paciente com fratura da coluna vertebral * p<0,05. AF: ânulo fibroso.

**Figura 4:** Níveis séricos de ácido hialurônico. Controle se refere a paciente saudável. Degeneração do disco se refere a paciente com degeneração do disco intervertebral. Fratura abrange paciente com fratura da coluna vertebral * p<0,05. AH: ácido hialurônico.

## DISCUSSÃO

A CatB é uma protease que acredita-se ter papel importante na degradação de proteínas de MEC de cartilagem, como agrecano e colágeno.^(^^10^^,^^12^^,^^18^^)^A expressão aumentada de CatB tem sido relatada em condrócitos de pacientes com osteoartrite.^(^^19^^)^Além disso, células degeneradas intervertebrais secretam citocinas pró-inflamatórias, incluindo IL-6.^(^^20^^)^A atividade pró-inflamatória persistente da IL-6 favorece o acúmulo de células mononucleares no local da lesão, a angioproliferação e funções antiapoptóticas de células T.^(^^21^^-^^23^^)^

Os dados sugerem que tanto a IL-6 quanto a CatB estão intimamente relacionadas à fisiopatologia da DDI. Em nosso estudo, a expressão da proteína CatB foi significativamente aumentada nos tecidos discais degenerativos em comparação com os discos de fratura. A imunorreatividade intensa para CatB foi encontrada nas MEC de espécimes de disco degenerativo, sugerindo possível microambiente acídico causado pelo processo inflamatório que favoreceu a secreção de CatB. Além disso, os níveis de proteína IL-6 foram maiores quando comparados aos discos intervertebrais do Grupo Controle.

Não foi encontrada diferença significativa da atividade de CatB e da IL-6 nos grupos quando os plasmas foram analisados. As concentrações circulantes de IL-6 e CatB em pacientes com fratura da coluna vertebral também foram significativamente maiores quando comparadas ao Grupo Controle, mostrando que o aumento de IL-6 e CatB no soro não é específico da DDI. O aumento da concentração sérica de IL-6 e CatB em pacientes com fratura pode ser devido à inflamação aguda causada pela fratura, especialmente se considerarmos que o sangue periférico foi coletado logo após o trauma.

As análises quantitativas da AH sérica são úteis no diagnóstico de várias doenças inflamatórias.^(^^16^^,^^24^^,^^25^^)^A degradação e o *turnover* da MEC resultam na liberação de fragmentos de AH e AH na circulação sistêmica.^(^^26^^)^Diferentemente, em pacientes após tratamento inflamatório, níveis baixos de AH são encontrados na circulação.^(^^23^^,^^26^^)^

Podemos sugerir que níveis aumentados de AH sugerem processo de degeneração. No entanto, outros estudos são necessários para confirmar essa indicação, uma vez que o presente estudo representa resultado preliminar.

Sabe-se que a degradação dos componentes da MEC no processo de DDI é modulada por várias enzimas proteolíticas, das quais as metaloproteinases da matriz (MMPs) e as agrecanases desempenham papel crítico na ativação de IL-6, IL-8, ciclo-oxigenase-2 (COX2), MMP1/13 e receptores do tipo Toll (TLR2).^(^^27^^,^^28^^)^

É importante mencionar que, ultimamente, várias estratégias que visam à regeneração do disco intervertebral têm sido utilizadas. Tais estratégias, com resultados promissores, e envolvem terapias celulares e biomateriais, como o arcabouço do AH.^(^^28^^,^^29^^)^

Os resultados podem contribuir para estudos adicionais, que permitam o uso dessas moléculas-alvo para o processo de DDI.

## CONCLUSÃO

Os níveis séricos de ácido hialurônico em pacientes com degeneração do disco intervertebral são significativamente maiores do que os de pacientes não afetados e com fratura da coluna vertebral. A interlecuina 6 e a catepsina B estão possivelmente relacionadas à fisiopatologia da degeneração do disco intervertebral. No entanto, não representam um biomarcador no plasma.
